# Conserved features in TamA enable interaction with TamB to drive the activity of the translocation and assembly module

**DOI:** 10.1038/srep12905

**Published:** 2015-08-05

**Authors:** Joel Selkrig, Matthew J. Belousoff, Stephen J. Headey, Eva Heinz, Takuya Shiota, Hsin-Hui Shen, Simone A. Beckham, Rebecca S. Bamert, Minh-Duy Phan, Mark A. Schembri, Matthew C.J. Wilce, Martin J. Scanlon, Richard A. Strugnell, Trevor Lithgow

**Affiliations:** 1 Department of Microbiology, Monash University, Clayton 3800, Australia; 2 Department of Biochemistry and Molecular Biology, Monash University, Clayton 3800, Australia; 3 Medicinal Chemistry and Drug Action, Monash Institute of Pharmaceutical Sciences, Monash University, Parkville 3052, Australia; 4 Department of Materials Engineering, Monash University, Clayton 3800, Australia; 5 Australian Infectious Diseases Research Centre, School of Chemistry and Molecular Biosciences, University of Queensland, Brisbane, Queensland 4072, Australia; 6 Department of Microbiology & Immunology, University of Melbourne, Parkville 3052, Australia

## Abstract

The biogenesis of membranes from constituent proteins and lipids is a fundamental aspect of cell biology. In the case of proteins assembled into bacterial outer membranes, an overarching question concerns how the energy required for protein insertion and folding is accessed at this remote location of the cell. The translocation and assembly module (TAM) is a nanomachine that functions in outer membrane biogenesis and virulence in diverse bacterial pathogens. Here we demonstrate the interactions through which TamA and TamB subunits dock to bridge the periplasm, and unite the outer membrane aspects to the inner membrane of the bacterial cell. We show that specific functional features in TamA have been conserved through evolution, including residues surrounding the lateral gate and an extensive surface of the POTRA domains. Analysis by nuclear magnetic resonance spectroscopy and small angle X-ray scattering document the characteristic structural features of these POTRA domains and demonstrate rigidity in solution. Quartz crystal microbalance measurements pinpoint which POTRA domain specifically docks the TamB subunit of the nanomachine. We speculate that the POTRA domain of TamA functions as a lever arm in order to drive the activity of the TAM, assembling proteins into bacterial outer membranes.

Bacterial pathogens rely on membrane biogenesis pathways to assemble the surface structures essential to host-pathogen interactions such as adhesion and host invasion. In the case of Gram-negative bacteria, the process of outer membrane assembly is dependent on the Omp85-family protein BamA^1^,^2^,^3^ and, for some membrane structures, requires the Omp85-family protein TamA^3^,^4^,^5^. TamA combines with the inner membrane protein TamB to form the translocation and assembly module (TAM) and deletion of the *tamA* or *tamB* gene in species of *Klebsiella*^6^, *Proteus*^7^, *Citrobacter*^4^,^8^ or *Salmonella*^4^ diminishes the virulence of these pathogens.

The archetypal Omp85 protein BamA forms the core of the β-barrel assembly machinery (BAM) complex^1^,^2^,^3^. BamA has a large periplasmic domain composed of five *po*lypeptide *tr*ansport *a*ssociated (POTRA) repeats, and the crystal structure of the BamA POTRA domain fragment initially suggested a curved, fish-hook structure^9^. Small angle X-ray scattering (SAXS), nuclear magnetic resonance spectroscopy (NMR) and protein crystallography showed that the POTRA domains of BamA are actually capable of considerable rotational and lateral flexibility^10^,^11^,^12^. In BamA, each of the five POTRA domains is very similar in overall structure, and each has a characteristic hydrophobic cleft proposed to bind and deliver substrate proteins for insertion into the outer membrane^9^,^10^,^11^. A mystery remains over how the activity of BamA is energized, given that there is no transmembrane potential across the outer membrane, nor access to nucleotide hydrolysis, as utilized in equivalent reactions at the inner (cytoplasmic) membrane^13^.

The crystal structure of TamA shows it to have an Omp85 C-terminal β-barrel domain and a set of three POTRA domains that project into the periplasm^5^. Previously, the three POTRA domains from TamA (*i.e.* TamA POTRA1–3) were considered analogous to the membrane-proximal POTRA domains of BamA (*i.e.* BamA POTRA3–5) and it was thereby suggested that TamA POTRA1–3 likewise functions in substrate binding by a process of beta-augmentation^5^. Recent magnetic contrast neutron reflectrometry (MCNR) analysis showed that the TamA subunit can catalyze autotransporter insertion into a reconstituted membrane environment; this coincides with a movement of the POTRA domain of ~30 Å relative to the membrane surface, and deletion of the entire POTRA domain from TamA prevented substrate protein insertion into the membrane^14^. Thus, some or all of the POTRA domains are required for the catalytic activity of TamA, and are also required for interactions between TamA and its partner protein TamB^4^. Presumably, there is a specific site where TamB makes its interaction with TamA, and determining how TamA and TamB interact is a necessary first step towards prospective sites of therapeutic intervention with a view to drug development strategies. Adding further to the complexity to our understanding of how BamA and TamA function is the recent observations that there are as many as ten distinct sub-families of Omp85 proteins^15^: within the Omp85 proteins classified as “BamA”, the number of POTRA domains appears to vary from one to seven^15^,^16^, over-lapping with the size of the TamA proteins, and for those proteins currently classified as “TamA” it seems that they are found only in Proteobacteria^15^.

In this report, we use CLuster ANalysis of Sequences (CLANS) to define that TamA POTRA domain sequences have characteristic sequence features and TamA proteins can therefore be classified with some certainty. We show that while TamA POTRA3 is similar to BamA-type POTRA domains, the other two POTRA domains of TamA differ significantly in their sequence and structural characteristics and provide a signature with which to distinguish TamA from other groups of Omp85 proteins. We used SAXS and NMR experiments to reveal the specific surface characteristics in these POTRA1 and POTRA2 domains of TamA, and show for the first time that the POTRA domains of TamA form a rigid body, distinct from the highly flexible arrangement seen in the POTRA domains of BamA proteins. We suggest a model that accounts for how the TAM can draw on the interaction between TamA and TamB to drive protein assembly into the outer membrane. Quartz crystal microbalance with dissipation monitoring (QCM-D) studies show TamA POTRA1 is essential to mediate this crucial interaction with TamB, thereby identifying a target for small molecule intervention to inhibit TAM function.

## Results

### Conserved sequence features in TamA from diverse bacteria

**Figure 1:**
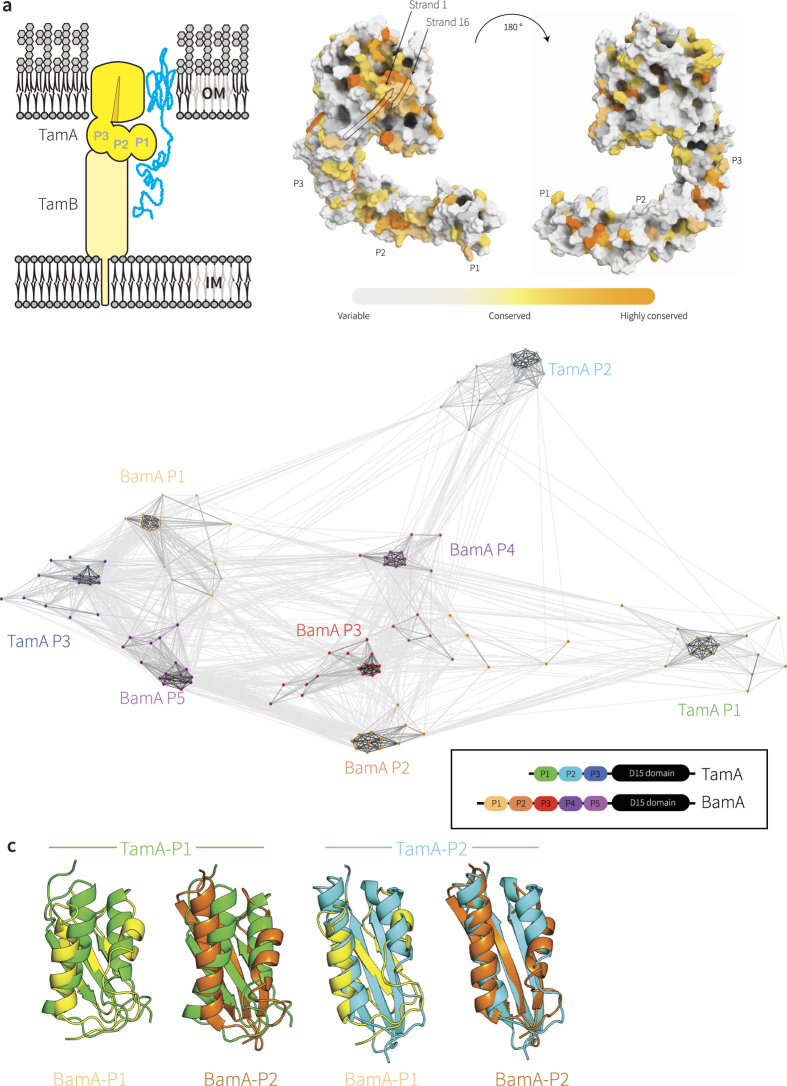
Selective pressure has maintained specific residues in TamA through the course of evolution. (**a**) Topology of TamA and TamB, spanning the bacterial outer membrane (OM) and inner membrane (IM). The TAM drives the insertion of substrate proteins (blue) such as Ag43 into the membrane^4^,^14^. Rate4Site analysis of TamA sequences (detailed in Supplementary Table 1) plotted onto the structural coordinates of TamA (pdb 4C00). The crenel in the β-barrel domain between the first and last β-strands is indicated with an arrow; POTRA domains are labeled P1, P2 and P3. (**b**) POTRA domain sequences from TamA and BamA of representative species (Supplementary Table 2) were extracted and subjected to CLANS^19^. Lines are shown connecting similar sequences with an P-value cut-off of 1e-2. Sequences derived from each POTRA domain form a distinct group, color-coded for the corresponding three TamA POTRA domains and the five BamA POTRA domains. (**c**) Representation of the LSQ fits between TamA POTRA 1 (green) and TamA POTRA 2 (cyan) and the BamA POTRA 1 (yellow) and BamA POTRA2 (orange), calculated using PyMOL (www.pymol.com/pymol). A complete comparison is shown in Supplementary Figure 2.

Rate4Site^17^ can be used to measure sequence conservation through evolution, and we mapped the Rate4Site scores onto the structure of TamA to highlight its highly-conserved features. The most highly-conserved face of the TamA β-barrel domain covers the lateral region where the first and last β-strands meet and the extension of β-strand 1 that forms an “exit pore” (Fig. 1a), with the equivalent region of BamA shown to be critically important for activity^12^,^18^, further supporting the suggestion that the β-barrel domain of BamA and TamA function by a similar mechanism^5^. Only two aromatic residues are among the conserved residues of TamA, and sequence logos were constructed to highlight these amino acids (Supplementary Fig. 1). The analysis revealed that the proposed active site of the β-barrel domain in TamA^5^ is characterized by a dearth of stabilizing aromatic residues amidst the otherwise highly-conserved sequences, in keeping with the mobility required by these strands according to the current models for the mechanism by which BamA and TamA act^12^,^18^,^5^. In addition, the Rate4Site analysis illustrated the conservation of discrete surface patches in TamA POTRA1 and TamA POTRA2 (Fig. 1a).

CLANS can be used to depict the similarity of amino acid sequences graphically, utilizing an all-against-all pairwise BLAST to cluster individual sequences. We subjected POTRA domain sequences of TamA and BamA from diverse bacterial species to CLANS to determine relationships conserved through evolution (Fig. 1b). In this plot, lines connect protein sequences with significant similarity and the length of each line is a function of how similar each sequence is to another^19^. The five POTRA domains from BamA are known to be highly similar in structure, with tertiary features that can be super-imposed with a root mean square deviation (r.m.s.d.) of 1.3–1.8 Å^11^. In a relative sense, the five POTRA domains from BamA are as similar to each other as they are to TamA POTRA3 (Fig. 1b, Supplementary Fig. 2), while TamA POTRA1 and TamA POTRA2 do not show such close relationship to the BamA POTRA domains (Fig. 1b). This is particularly evident in the placement of the alpha-helices that pack against the beta-sheet of the POTRA domains (Fig. 1c; Supplementary Fig. 2). We conclude that while the barrel domains of BamA and TamA proteins are homologous; the POTRA domains are homologues which have undergone very strong sequence divergence, or were derived by horizontal gene transfer from unknown donors.

### Solution structures of the TamA POTRA domains

**Figure 2:**
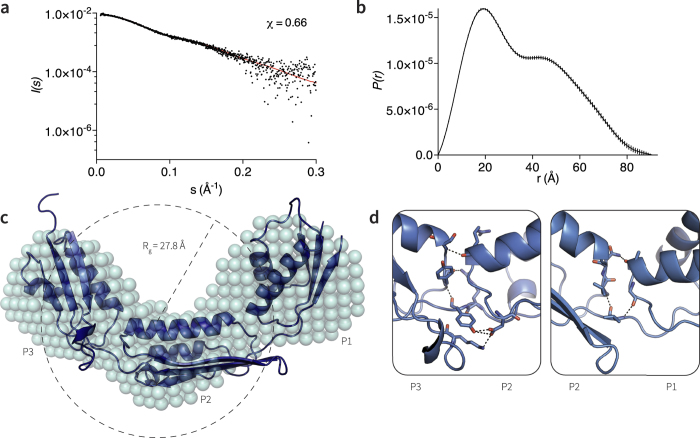
Solution small angle X-ray scattering structure of TamA POTRA1–3. (**a**) Scattering data fitted (black dots) fitted against predicted theoretical scattering curve using CRYSOL (red line). Calculated parameters shown in Supplementary Table 3. (**b**) Comparison of the pair-wise distribution *P*(*r*) functions calculated from the experimental and theoretical profiles plotted against *r* in Å (error bars are indicated). (**c**) Model derived from *ab initio* reconstruction, calculated using DAMMIF^39^ and DAMAVER^40^, as shown by the teal spheres. Superimposed on this is the crystal structure (pdb 4C00). (**d**) Representation of the interfaces between TamA POTRA2 and POTRA3 (left), and TamA POTRA1 and POTRA2 (right), revealing polar interactions between the POTRA domains. Drawn as sticks are the main interacting residues between each POTRA domain; dashed lines represent hydrogen bonds.

The POTRA domains of BamA show rotational flexibility in solution^10^,^11^,^20^, and this conformational freedom is also seen in distinct arrangements in crystal forms of the POTRA domains in BamA proteins from several species of bacteria^9^,^11^,^12^. By contrast, a comparison of theoretical scattering curves and experimental small angle X-ray scattering (SAXS) curves for TamA POTRA1–3 (Fig. 2a) showed good agreement for a rigid body model of similar shape to that seen in the crystallized conformation of TamA reported by Gruss *et al.*^5^. The pairwise distribution function (*P*(*r*)) calculated from the scattering curves (Fig. 2b) shows a bimodal profile suggesting the presence of more than one lobe in the shape of the molecule. The SAXS data were used to generate a structural model of TamA POTRA1–3, demonstrating that the solution structure is largely consistent with the size and shape of the crystal structure (Fig. 2c). Guinier analysis determined a 27.98 Å radius of gyration (*R*_g_). Examination of the inter-domain hydrogen bonds in the crystal structure of TamA highlighted numerous contacts between the POTRA domains that might explain the experimentally-determined rigidity (Fig. 2d). Previous solution studies on membrane-embedded TAM, using MCNR, defined movements of the POTRA domain relative to the membrane in the functional TAM^14^; the rigidity of the POTRA domain observed here by SAXS provides experimental support for a lever-arm mechanism for TAM function.

The shape of the SAXS model also highlighted that TamA POTRA2 is elongated, thereby displaying a larger surface area for protein-protein contacts compared to BamA POTRA domains (Supplementary Fig. 2). This is largely due to an unusual twist that alters the alignment of the two helices in this POTRA domain (Fig. 2c). Initial analysis of POTRA1 suggested a similar elongation and increase in surface area, however elements of the domain were not resolved in the crystal structure (pdb 4C00). We therefore expressed and purified POTRA1 to determine its structure by NMR, and this did indeed reveal a surface area of 8300 Å^2^, considerably larger than that found for the average POTRA domains from BamA (*e.g.* 7700 Å^2^; Supplementary Fig. 2).

**Figure 3:**
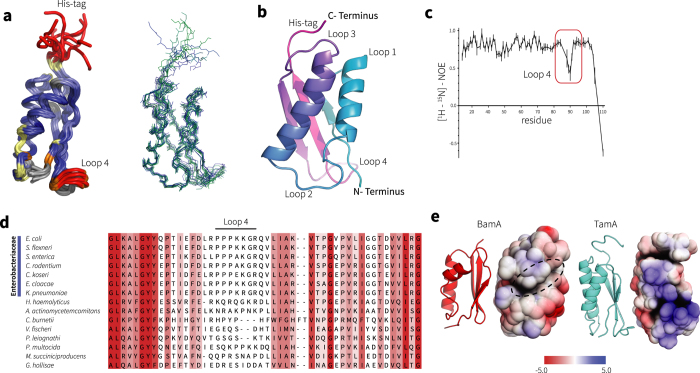
The POTRA1 and POTRA2 domains of TamA are structurally specialized. (**a**) NMR solution structure of TamA POTRA1, with backbone representation of the ensemble of the 10 lowest energy structures. Steady-state ^1^H-^15^N NOE values are color plotted onto a cartoon ribbon representation of the structure (Red: Lower values corresponding to high mobility, Blue: Higher values corresponding to low mobility). NMR data is detailed in Supplementary Table 4. (**b**) The closest to average structure of POTRA1. (**c**) Heteronuclear ^1^H-^15^N NOE measurements of the POTRA1 domain demonstrate the inherent flexibility of the loop, with the average value for loop 4 residues 88–91 being 0.45 ± 0.11 compared to the domain average of 0.77 ± 0.19. (**d**) Multiple sequence alignment in the region corresponding to loop 4. The amino acid composition in this loop from other Gamma-proteobacteria is consistent with a disordered structure seen in the *E. coli* protein. (**e**) Surface view of the surface charge features of BamA POTRA1 and TamA POTRA1 created between the two helices (red and blue surfaces represent acidic and basic residues, respectively).

Views of the 10 lowest energy structures generated from NOE-based distance constraints and dihedral angle constraints derived from secondary chemical shifts and refined in water (Fig. 3a) reflect that the solution structure is well defined with a backbone heavy atom r.m.s.d. of 0.67 Å (Supplementary Table 4 shows the structural statistics for the ensemble). The closest to average structure (Fig. 3b) highlighted two distinguishing features of TamA POTRA1: (i) a highly-elongated shape accentuated by a dynamic segment of the polypeptide (loop 4) between the second and third β-strands (Fig. 3c), with the length of this loop largely conserved across bacterial species (Fig. 3d), and (ii) a displacement in two α-helices that increases the overall surface area (Supplementary Fig. 2) and created a basic groove surface feature on one face (Fig. 3e). This is in contrast to the hydrophobic groove found on the equivalent face of all five POTRA domains from BamA that were proposed to form the substrate binding site^11^. These obvious differences in structure taken together with sequence divergence from BamA POTRA domains strongly suggest that TamA POTRA1 fulfils a distinct biological role.

### TamA POTRA1 binds TamB

**Figure 4:**
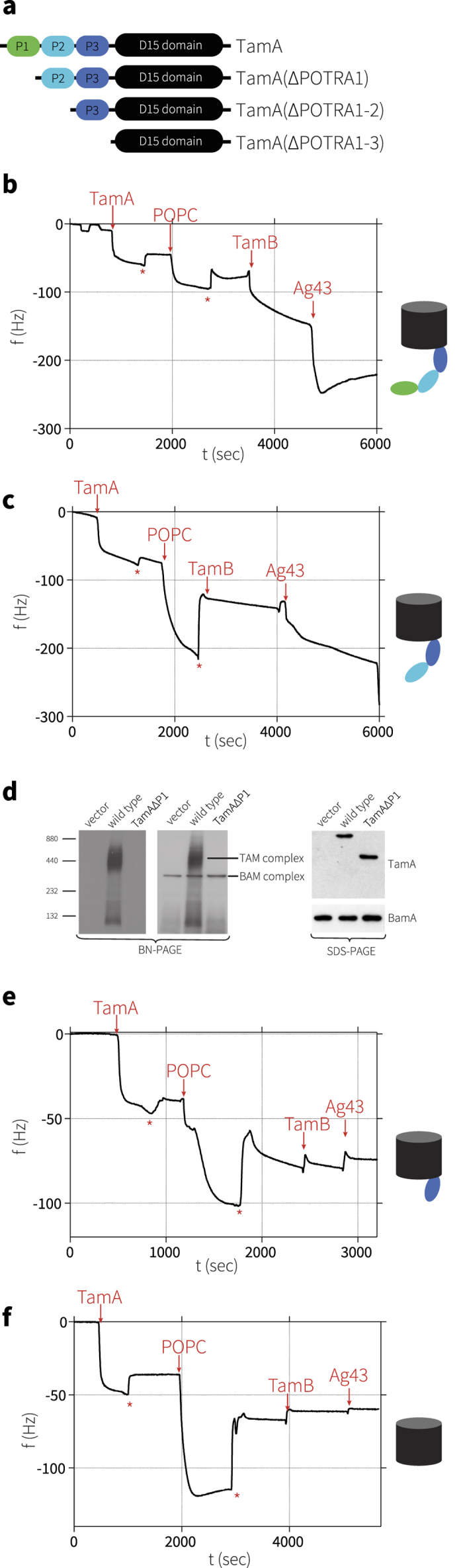
Specific functions for the POTRA domains of TamA. (**a**) Cartoon depicting the POTRA deletion series of TamA. The purification of these proteins from detergent-solubilized membranes is detailed in Supplementary Fig. 3. (**b**) QCM-D measurements show the frequency response, indicative of mass changes, to (1) the attachment of TamA to the gold-surface, (2) reconstitution of the membrane layer with phospholipids, (3) the addition of purified TamB, and (4) addition of urea-denatured Ag43. (**c**) QCM-D measurements for TamA(ΔPOTRA1). (**d**) Membranes were isolated from *E. coli* expressing TamA or TamA(ΔPOTRA1) and subject to BN-PAGE or SDS-PAGE and analysis by immunoblotting. **(e)** QCM-D measurements for TamA(ΔPOTRA1–2). **(f**) QCM-D measurements for TamA(ΔPOTRA1–3). Asterisks (*) – wash step (with 20 mM TRIS and 150 mM NaCl). Replicate experiments are presented in Supplementary Figure 4.

Since TAM function is required for pathogenesis, and this function is ablated with the loss of either TamA or TamB^4^, the interaction between TamA and TamB is crucial *in vivo.* To address which TamA POTRA domain is required for the docking of TamB, a deletion series of truncated TamA proteins (Fig. 4a) was assessed in a QCM-D assay system with a histidine-tagged form of TamA attached to a gold surface with a membrane layer reconstituted around the protein^14^. QCM-D is an extremely sensitive technique that allows for accurate, temporal based monitoring of binding to a surface by measuring sub-nanogram mass changes. This technique is preferable to examine changes in membrane embedded protein systems as it measures the system as a whole, including trapped solvent and changes in lipids.

After addition of TamB, a specific binding event was measured as a change in frequency that is directly related to the increased mass on the surface (Fig. 4b, step 3), demonstrating the formation of the TAM. In contrast, TamB failed to bind to TamA(ΔPOTRA1) demonstrating the importance of the POTRA1 domain in the interaction between TamA and TamB (Fig. 4c, step 3). To confirm that, *in vivo*, TamB binding depends on the POTRA1 domain of TamA, membranes were isolated from *E. coli* strains expressing TamA with or without the POTRA1 domain, and analyzed by blue-native gel electrophoresis (BN-PAGE). The absence of TamA POTRA1 prevented formation of a TAM (Fig. 4d).

Using either QCM-D or neutron reflectometry assays, the presence or absence of TamB has no influence on whether substrates such as Ag43 bind to TamA^14^. We therefore tested each of the deletion constructs for their ability to bind the substrate protein Ag43. As previously reported, Ag43 was bound by the membrane-embedded TamA, as judged by a rapid decrease in frequency (Fig. 4b, step 4 “Ag43”). TamA(ΔPOTRA1) also bound Ag43, albeit at a substantially slowed rate (Fig. 4c, step 4 “Ag43”). In contrast, TamA(ΔPOTRA1–2) failed to bind Ag43 (Fig. 4e, step 4 “Ag43”), demonstrating that the TamA β-barrel domain and POTRA3 are unable to effectively capture the substrate, and showing that interaction of Ag43 with the reconstituted membrane is TamA-dependent. Consistent with this, TamA(ΔPOTRA1–3) also failed to bind Ag43, as judged from no change in frequency after addition of Ag43 (Fig. 4f, step 4 “Ag43”).

## Discussion

The Omp85 family of proteins contribute variously to protein secretion systems and membrane protein assembly machinery^1^,^2^,^3^. TamA and BamA are Omp85-family proteins that function in parallel pathways for outer membrane protein assembly in bacteria, and each has similar domain architecture. The β-barrel domain of BamA and TamA appear to function through a conserved mechanism: structural analysis of both proteins defined a juncture between the first and last strands of the β-barrel domain as a site of substrate binding and folding^5^,^12^,^18^,^21^,^22^, and this region of TamA shows structural conservation with BamA^5^. We found here by Rate4Site analysis that the specific region around the first and last strands of the TamA barrel-domain is the most highly-conserved feature found across TamA orthologs, in accordance with the proposal of its functional importance^5^. A detailed analysis of the POTRA domains of TamA revealed why it is that these alone can be used to distinguish the BamA- and TamA-subfamilies of Omp85 proteins.

Crystallization of BamA revealed at least two conformations of the POTRA domains^9^,^10^,^11^,^12^,^23^, and SAXS analysis of POTRA domains shows flexibility in this region of BamA ^10,11^. Genetic analysis and limited proteolysis^24^,^25^ has suggested that switching between conformational states is driven by the movements of the BamD subunit^25^ and BamB against the BamA subunit^23^. Given the size and complex topology of the BamD, BamC, BamB and BamE partner subunits of BamA, the inter-POTRA domain flexibility might assist the OM partners to be re-organized to mediate these conformational shifts. It remains unclear, however, what source of energy the BAM complex accesses in order to drive protein insertion into the outer membrane.

A different mechanism is employed to drive the activity of the TAM. Unlike the BAM complex, the TAM includes a subunit that is anchored in the bacterial inner (cytoplasmic) membrane. This TamB subunit contains an N-terminal signal-anchor transmembrane segment^4^, extends in an elongate form across the periplasmic space^14^, and contacts selectively with the POTRA domains of TamA (this study). Here we have documented the distinguishing features of TamA, defined the interaction site between TamA and TamB, and rationalized a mechanism by which TamB could regulate conformational changes in TamA to drive its function in outer membrane protein assembly.

**Figure 5:**
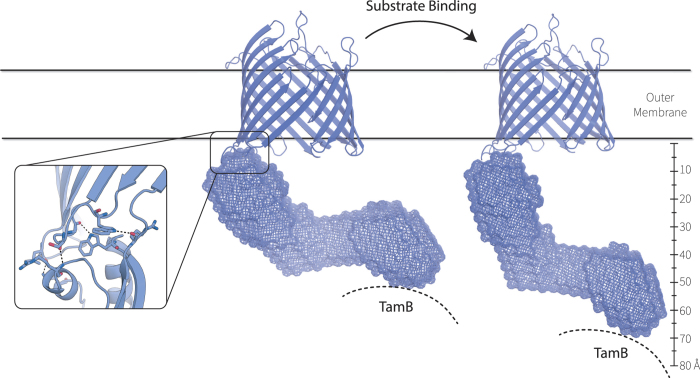
Molecular blueprint of the TAM nanomachine. A composite structure of TamA, incorporating information from the NMR structure of POTRA1 (pdb 2LY3) and the SAXS structural information superimposed as a meshwork onto the crystal structure (pdb 4C00). The β-barrel domain is represented as a ribbon diagram for clarity, and the inset details the contacts between residues in the POTRA3 domain and the β-barrel domain, as previously documented by Gruss *et al.*^5^. Given the discovery that TamA POTRA1 mediates contact with TamB, the dotted line denotes where TamB applies resistance to the TamA lever arm. The scale bar demonstrates the extent of movement that the TamA lever is capable of, given MCNR measurements^14^ showing that it can extend to ~77 Å from the internal surface of the outer membrane in response to the presence of a substrate protein. This movement would necessarily require rearrangement of the POTRA3:β-barrel domain contacts, and we speculate that restoring these contacts could be a basis for bringing the POTRA domain lever arm back to the resting position, for continued rounds of protein assembly *in vivo*.

The POTRA domains of TamA have distinguishing structural characteristics: they lack the conformational flexibility seen in BamA, and instead adopt a conformation in solution that is consistent with what we propose to be a lever-arm in the TAM. In SAXS measurements, less than 10 Å movement was possible between POTRA1 and POTRA2, and none was observed between POTRA2 and POTRA3. This is consistent with extensive crystal contacts observed between the POTRA domains in TamA^5^. Taken together, this suggests that major movements, measured previously at ~30 Å relative to the membrane surface^14^ would be contributed through a fulcrum at the POTRA3:β-barrel domains (Fig. 5).

In addition, the POTRA1 and POTRA2 domains of TamA both have increased surface areas including highly-conserved residues, compared to the general features in the POTRA domains of BamA. TamA POTRA1 showed a defined surface of conserved sequence characteristics (Rate4Site), sequence-based distinguishing features (CLANS) and structural differences (LSQ) that contribute to its essential role in the docking of TamB to TamA to form the functional TAM. TamA POTRA2 also showed sequence characteristics that distinguish it from the other POTRA domains analysed, and a conserved face of the POTRA1-POTRA2 region of TamA could function in docking to TamB. Any model for how the TAM functions, must account for how the TamA-TamB interaction enables function: deletion of the *tamB* gene phenocopies deletion of the *tamA* gene^4^. We suggest that TamB provides the resistance needed for the TamA lever arm to work in the context of driving outer membrane protein integration (Fig. 5). Defining POTRA1 as a major point of contact mediating this interaction is important, in that it identifies a target for small molecule intervention that would break the interface between TamA and TamB, and could potentially be developed for anti-infective therapy. In this regard too, we note with interest the recent report of crystallization of the highly-conserved C-terminal (DUF490) domain of TamB^26^, opening an avenue to future research aimed at understanding for why the loop in POTRA1 is conserved and how the conserved face of POTRA1 and POTRA2 sits relative to the DUF490 domain of TamB.

## Methods

### Culture methods and bacterial strains

Bacterial strains, plasmids and oligonucleotide primers used in this study are detailed in Supplementary Tables 6, 7 and 8, respectively.

### Membrane protein isolation and analysis

Total bacterial membranes were harvested from overnight cultures supplemented with 10 mM IPTG, chloramphenicol and ampicillin according to previously described methods^27^ and total protein amount was equalized according to the A_280 nm_ measurement taken after solubilization in 0.06% sodium dodecylsulphate (SDS). Total membrane proteins were analyzed by SDS-PAGE and immunoblotting using anti-BamA (1:10,000), anti-TamA (1:5,000) and anti-TamB (1:10,000) antisera^4^. Native protein complexes were solubilized from total bacterial membranes with 1% ndodecyl-βDmaltoside (DDM) then analyzed by BN-PAGE as previously described^27^.

### Sequence analysis

For the clustering representation of the POTRA domains of BamA and TamA homologues, protein sequences were manually selected (accession numbers detailed in Supplementary Table 2). Alignment of the sequences was performed with muscle^28^ as implemented in Seaview (version 4.3.3 29), and POTRA domain boundaries were determined based on the *E. coli* TamA sequence as defined in the crystal structure^5^ (POTRA1: 26–102, POTRA2:106–186, POTRA3:190–261) and for BamA according to the start or end positions of the respective β-strand as given in the protein databank (PDB) summary for *E. coli* BamA (http://www.rcsb.org/pdb/protein/P0A940; POTRA1: 24–90, POTRA2: 93–171, POTRA3: 176–261, POTRA4: 267–342, POTRA5: 348–419). CLANS software^19^ was used under default conditions, and the connections used correspond to a P-value cut-off of 1e-02.

### QCM-D analysis

A Q-SENSE E4 system equipped with an Axial Flow Chamber (chamber volume is 2.5–3.5 μl, with a 10 μl dead volume) was used and a constant temperature was set 25 °C during the experiments. Surface modification of the Ni-ANTA functional group on the gold surface was carried out according to published protocols^14^. Modified NTA gold sensor crystals were mounted in the chamber and the change in frequency of the sensor movement (Δ*f*) and dissipation (ΔD) were conducted. Before adding protein samples to the cell, the ANTA-modified surface was incubated with a 100 mM NiCl_2_ solution to charge the Ni^2+^ via the carboxylates group of ANTA. Subsequently the surface was rinsed with water and then 20 mM TRIS/150 mM NaCl/0.03% DDM solution, pH 7.5. a 1.0 mL volume of 0.1 mg/ml protein solution was then injected into the cells using a flow pump with a flow velocity of 200 μl/min.

Protein concentrations were determined at 280 nm using a NanoDrop spectrophotometer, and a 1.0 mL volume of 0.1 mg/ml of detergent-solubilized TamA, TamA(ΔPOTRA1), TamA(ΔPOTRA1–2 or TamA(ΔPOTRA1–3) was introduced into the chamber (step1). After protein adsorption, the surface was rinsed with at least 20 chamber volumes of buffer without DDM before reconstituting a lipid bilayer as previously documented^14^. The quality of these reconstituted bilayers has been validated by neutron reflectrometry^14^. The supported bilayer was formed by phospholipid (POPC) coadsorption with β-D-dodecylmaltoside (DDM) with a molar ratio of 1:6 at final concentration of 1 mg/ml in buffer followed by further adsorption from 10 to 100 times diluted solution^14^. After attaining equilibrium, the surface was rinsed with at least 20 chamber volumes of buffer (~0.5 mL) without detergent to remove DDM residuals. Following that, 0.5 mL of 0.2 mg/ml TamB and 0.5 mL of 0.2 mg/ml Ag43 were subsequently added into the flow chamber, shown as (step 3) and (step 4) respectively, followed by the washing procedures described above.

### Plasmids to express TamA(ΔPOTRA1), TamA(ΔPOTRA1–2) and TamA(ΔPOTRA1–3)

The template for these POTRA deletion constructs was a codon-optimized *tamAB* gene cloned into pBADHisA (Invitrogen, UK) to generate pBADTamAB^14^. To construct pUC57TamABΔP1 and pUC57TamABΔP1–2, 458- and 377- bp fragments, respectively, were synthesized *de novo* and cloned into pUC57. pUC57TamABΔP1 and pUC57TamABΔP1–2 were digested separately with MluI and EcoRI, MluI and BstBI, and with MluI and SpeI, and subcloned into pBADTamAB-C-term-6xHis, predigested with the same restriction enzymes, to create mutants with one, two, and three POTRA deletions: pBADTamAB-C-term-6xHisΔP1 and pBADTamAB-C-term-6xHisΔP1–2, respectively. To construct pBADTamA-C-term-6xHis, pBADTamA-C-term-6xHisΔP1 and pBADTamA-C-term-6xHisΔP1–2, PCR was performed on 500 ng of template DNA (pBADTamAB-C-term-6xHis) with 1 μg each of primers TamAMluIFor (5′-GAAACGCGTAACAAAAGTGTCTATAATCACG-3′) and TamAHindIIIRev (5′-CAGCCAAGCTTTCATAATTCTGGCCCCAGA-3′). Amplicons and target vector (pBADHisA) were then digested with MluI and HindIII, and ligated. Construction of the plasmid for expression of TamA(ΔPOTRA1–3) was described previously^14^.

### Purification of TamB, TamA(ΔPOTRA1), TamA(ΔPOTRA1–2) and TamA(ΔPOTRA1–3)

Ag43 was purified as previously described^14^. Protein expression used cultures in LB with ampicillin (100 ug/ml). TamB was produced and purified without the N-terminal transmembrane segment as described^14^; briefly, BL21(DE3)* cells were transformed with pET22b plasmid encoding TamB. These cells were grown in Luria Broth at 30 °C until an OD600 of 0.6–0.7 whereupon Isopropyl β-D-1-thiogalactopyranoside (IPTG) was added to the culture to a final concentration of 0.1 mM. The culture was grown for a further 12 hours, cells were then harvested by centrifugation at 8000 g for 10 minutes at 4 °C. The cell pellet was resuspended in lysis buffer (20 mM Tris.HCl, pH = 8.0, 150 mM NaCl, 5 mM MgCl_2_); DNaseI (20 μg/mL) and lysozyme (1 mg/mL) was added and the resultant mixture was incubated at 4 °C for 30 minutes. The resuspended cells were ruptured by high-pressure emulsification using an Avestin C5 cell disruptor. Cell lysate was clarified by centrifugation at 20,000 g for 15 minutes at 4 °C. The clarified supernatant was than passed through a Nickel immobilised metal affinity column (GE Lifesciences, 5 mL HisTRAP-HP), the bound protein was washed and subsequently eluted using an increasing imidazole gradient. Fractions containing TamB were pooled and concentrated in a centrifugal membrane concentrator, and were immediately subjected to size exclusion chromatography with a buffer containing 20 mM Tris.HCl, pH = 7.5, 150 mM NaCl (Supplementary Fig. 3). Fractions containing TamB were pooled and used as eluted for QCM experiments.

The TamA proteins were over-produced in Top10 cells by growing cultures at 30 ^o^C to an OD600 of 0.6–0.7. The growth temperature was switched to 16 ^o^C. Expression was induced by addition of arabinose to 0.01%, and culture continued overnight. Cells were harvested by centrifugation at 8,000 g for 10 min at 4 ^o^C. The pellet was resuspended in lysis buffer (20 mM TRIS, pH 7.5, 150 mM NaCl and PMSF) and a protease inhibitor cocktail was added before disrupting the cells in an Avestin cell disruptor. Unbroken cells were removed by centrifugation at 26,000 g for 15 min. The supernatants were ultracentrifuged at 142,000 g at 4 ^o^C for 60 min to isolate the membrane fraction. The membrane pellets were resuspended in 20 mM TRIS/150 mM NaCl/5% Elugent, pH 7.5 and were incubated overnight at 4 ^o^C to solubilize membrane proteins. The supernatants were then applied to 1 ml Ni-NTA affinity columns and were pumped through the column overnight (~16 hours). The columns were then washed with 10 ml washing buffer (20 mM TRIS/150 mM NaCl/20 mM imidazole/0.5% Elugent, pH 7.5), and, for protein elution, 20 mM TRIS/150 mM NaCl/500 mM imidazole/0.5% Elugent, pH 7.5 was used. Proteins were subject to size exclusion chromatography in 20 mM TRIS, 150 mM NaCl and 0.5% Elugent to remove aggregated proteins (Supplementary Fig. 3). The soluble protein fraction was then dialysed with 20 mM TRIS, 150 mM NaCl and 0.03% DDM buffer solution.

### Purification of the TamA POTRA1 domain

A plasmid encoding recombinant TamA POTRA1 domain (residues 22–104) was constructed by amplifying the desired coding region of pCM6 using the primer pair 0233a and 0233b (See Supplementary Table 8) with NEB-*Phusion* polymerase. The PCR fragment encoding TamA POTRA1 was then inserted into the plasmid pET-21d, using the *Nco*I and *Xho*I sites, to create plasmid pMB1. pMB1 was transformed into *E. coli* BL21-DE3* (Novagen), grown in LB broth at 30°C and, when the culture reached an optical density (600 nm) of 0.4, IPTG was added to a final concentration of 1 mM. Cells were harvested 19 hours after IPTG induction by centrifugation, resuspended in 25 mL buffer per litre of medium (20 mM Tris.Cl pH 8.0, 500 mM NaCl, 30 mM Imidazole) and lysed using an Emulsiflex (3 passes at 15,000 psi). The cell lysate was clarified by centrifugation at 20,000 *x g* for 15 minutes and the supernatant was purified by Ni^2+^ affinity chromatography using a GE^TM^-HisTrapFF (5 mL) column. The protein was eluted as per manufacturers instruction, with the wash and elution buffers containing (20 mM Tris.Cl pH 8.0, 500 mM NaCl, 30 mM Imidazole) and (20 mM Tris.Cl pH 8.0, 500 mM NaCl, 400 mM Imidazole) respectively. After elution, the TamA POTRA1 protein was concentrated in a centrifugal concentrator and applied directly onto a Superdex^TM^-75 (16/60) (GE-Lifesciences) column which had been equilibrated with buffer (20 mM NaH_2_PO_4_, pH = 7.4, 50 mM NaCl). After this size-exclusion chromatography (SEC) step, fractions were analyzed by SDS-PAGE and high-resolution electrospray mass-spectrometry to assess purity. In a modification to this protocol, a ^15^N and ^13^C double-labelled sample was prepared by growing BL21-DE3* cells in M9 minimal media containing ^13^C-labelled glucose and ^15^N-labelled ammonium chloride, according to a previously described method^30^.

### Structural determination

NMR experiments were performed using 1.8 mM TamA POTRA1 in 95% H_2_O/ 5% ^2^H_2_O containing 20 mM Na_2_HPO_4_ pH 6.4, 50 mM NaCl, 0.5 mM EDTA. The majority of spectra were recorded on a 600 MHz Bruker AVANCE spectrometer using a triple resonance CryoProbe™. The following 2D spectra were acquired with conventional sampling: ^15^N-HSQC, constant time (26 ms) aliphatic ^13^C-HSQC, (HB)CB(CGCD)HD, (HB)CB(CGCDCE)H and ^15^N-NOESY-HSQC (tm 120 ms). Non-uniform sampling was used to record HNCACB, HNCO, HN(CA)CO, CBCA(CO)NH, H(CCCO)NH and (H)C(CCO)NH TOCSYs (tm 12 ms) and HBHA(CO)NH spectra. Aliphatic (tm 75 ms) and aromatic ^13^C-NOESY-HSQCs (tm 120 ms) were acquired on an 800 MHz Bruker AVANCE spectrometer using a triple resonance CryoProbe™. A ^1^H-^15^N NOE was acquired at 600 MHz on a Varian INOVA spectrometer fitted with a cryogenically cooled probe. For the NOE experiment 4 s saturation was used during the recycle delay and the spectrum was compared to one acquired without saturation during the 4 s recycle delay. NMR data were processed in TOPSPIN version 3.0 (Bruker Biospin™). The processed spectra were analysed using CARA software using MATCH and ASCAN algorithms for backbone and side-chain assignments, respectively.

Automated backbone assignments were made by the program MATCH using HNCACB and CBCA(CO)NH spectra. Missing assignments were assigned manually with reference to the HNCO and HN(CA)CO spectra. H^α^ and H^β^ assignments were made using the HBHA(CO)NH spectrum. ASCAN achieved ~75% of other sidechain ^1^H and ^13^C resonances using the 3D aliphatic ^13^C-NOESY-HSQC and ^15^N-NOESY-HSQC spectra. These were validated and the majority of the remaining resonances assigned using the H(CCCO)NH and (H)C(CCO)NH TOCSY spectra. Aromatic residues were assigned using the (HB)CB(CGCD)HD, (HB)CB(CGCDCE)H and aromatic ^13^C-NOESY-HSQC.

Near complete assignments were made for non-labile residues of TamA POTRA1. The exceptions were all resonances of the N-terminal residue Ala22, the backbone amides of residues Asn23 and Arg55 and the HG resonances of Leu26 and Leu103. Side-chain amides were able to be assigned for Gln42 and Gln92 based on NOE connectivity. ^1^H, ^13^C, and ^15^N chemical shifts assignments have been deposited in the BioMagResBank database (BMRB)^31^ with the accession number 18707.

Automated NOE cross peak picking and structure determination were performed with the AtnosCandid program^32^,^33^ using the CNS^34^ simulated annealing algorithm. Initial structures were generated from an extended strand conformation using simulated annealing with torsion angle dynamics for the high temperature and fast cooling stages followed by Cartesian dynamics for a second slow cooling stage. The AtnosCandid program incorporated 70 dihedral restraints derived from the C^α^ and C^β^ chemical shifts. These initial structures were used as starting structures for a second round of structure generation in AtnosCandid using Cartesian dynamics for both the high temperature and cooling phases. During this phase 143 backbone dihedral restraints generated from TALOS^35^ were included. The structures were refined in CNS with hydrogen bond restraints added for residues pairs with a unique pattern of hydrogen bonding in regions of canonical secondary structure as determined by structural convergence. The 10 lowest energy structures with no NOE violations >0.3 Å, bond violations >0.05 Å and no angle, improper or dihedral violations >5° were selected to represent the solution structure of TamA POTRA1.

### LSQ structural alignments and vacuum electrostatics

The vacuum electrostatic representation of TamA POTRA1 domain was calculated using the Adaptive Poisson-Boltzmann Solver^36^ and visualised by the PyMOL software package (http://www.pymol.org/pymol). LSQ structural alignments (Fig. 1c, Supplementary Fig. 2) were calculated using the PyMOL ‘super’ and ‘CEalign’ subroutines. LSQ structural alignments of TamA POTRA domains with BamA POTRA domains are detailed in Supplementary Table 5.

### Size exclusion-coupled small angle X-ray scattering

The plasmid encoding TamA POTRA1–3 was constructed by amplifying the desired coding region of pCM6 using the primer pair 0214 and MB114 (See Supplementary Table 8) with NEB-*Phusion* polymerase. The PCR fragment was then inserted into the plasmid pPROEX-Htb, using the *Nco*I and *Xho*I sites, to create plasmid pMB16. The plasmid was transformed into C41(DE3) OverExpress^TM^ (Lucigen) and the protein was purified in a similar manner to pMB1, with the exception that after initial affinity purification the protein was subjected to TEV protease (Novex) treatment for 12 hours at 4 °C, where upon it was subjected to another round of affinity purification to remove the cleaved affinity-tag peptide and TEV protease. The resulting mixture underwent two rounds of SEC (Sephadex-75) in a phosphate buffer ([KHPO_4_] = 50 mM (pH = 7.5), [NaCl] = 250 mM, Glycerol = 10% v/v). Fractions containing the protein were concentrated in a centrifugal filter to a concentration of 10 mg/mL and were immediately analysed by SEC-SAXS.

SEC-SAXS data were collected at the Australian Synchrotron SAXS/WAXS beamline using an inline Shimadzu Prominence Modular HPLC coupled to a SEC column with a pore size of 150 Å (WTC015s5, Wyatt Technology Corporation Pty Ltd). A 100 μl injection containing 1040 μg of TamA POTRA1–3 was subjected to SEC-SAXS in SEC buffer at a flow rate 0.2 ml/min, through a 1 mm quartz capillary held in the X-ray beam at 10 °C. Scattering data were collected from the eluent across at least one column volume using X-ray exposures at 0.05 second intervals. Data were collected using a Pilatus 1 M detector at a distance of 2.3 m, covering a *q* range of 0.007 to 0.35 Å^-1^. Primary data reduction was performed using the Scatterbrain software (Australian Synchrotron). SAXS data giving consistent radius of gyration (*R*_g_) values, and corresponding to the middle of the SEC peak of the 280 nm absorbance profile were averaged. The *I*(0) and *R*_g_ values were determined using Guinier approximation^37^. The *P*(*r*) and *R*_max_ were calculated using GNOM^38^ and theoretical scattering curves were predicted and fitted using CRYSOL^39^. Fifty *ab initio* models were generated using DAMMIF^40^. Using DAMAVER^41^ 2 models were excluded as outliers, the remaining 48 were averaged, filtered and superposed. PyMOL was use for three dimensional graphics representations. The *R*_max_ and *R*_g_ values were calculated for the X-ray crystal derived structure (PDB; 4C00) using CRYSOL^39^.

## Additional Information

**How to cite this article**: Selkrig, J. *et al.* Conserved features in TamA enable interaction with TamB to drive the activity of the translocation and assembly module. *Sci. Rep.* **5**, 12905; doi: 10.1038/srep12905 (2015).

## Supplementary Material

Supplementary Information
