# Supplementary material for: Conserved features in TamA enable interaction with TamB to drive the activity of the translocation and assembly module
Source: Sci Rep. 2015 Aug 5;5:12905. doi: 10.1038/srep12905 (PMC4525385; doi:10.1038/srep12905)
Supplement: Supplementary Information [file srep12905-s1.pdf]

Conserved features in TamA enable interaction with TamB to drive the activity of the translocation and assembly module.

Joel Selkrig[1,2,#,+], Matthew J. Belousoff[1,+], Stephen J. Headey[3,+], Eva Heinz[1], Takuya Shiota[1], Hsin-Hui Shen[1,4], Simone A. Beckham[2], Rebecca S. Bamert[1], Minh-Duy Phan[5], Mark A. Schembri[5], Matthew C.J. Wilce[2], Martin J. Scanlon[3], Richard A. Strugnell[6] and Trevor Lithgow[1]*

1 - Department of Microbiology, Monash University, Clayton 3800, Australia

2 - Department of Biochemistry and Molecular Biology, Monash University, Clayton 3800, Australia

3 - Medicinal Chemistry and Drug Action, Monash Institute of Pharmaceutical Sciences, Monash University, Parkville 3052, Australia

4 - Department of Materials Engineering, Monash University, Clayton 3800, Australia

5 - Australian Infectious Diseases Research Centre, School of Chemistry and Molecular Biosciences, University of Queensland, Brisbane, Queensland 4072, Australia

6 - Department of Microbiology & Immunology, University of Melbourne, Parkville 3052, Australia

+ - These authors contributed equally; # - Present address: European Molecular Biology Laboratory, Genome Biology Unit, 69117 Heidelberg, Germany; * - Correspondence: trevor.lithgow@monash.edu

This file includes:

Supplementary Figures 1-4
Supplementary Tables 1-8
Supplementary References

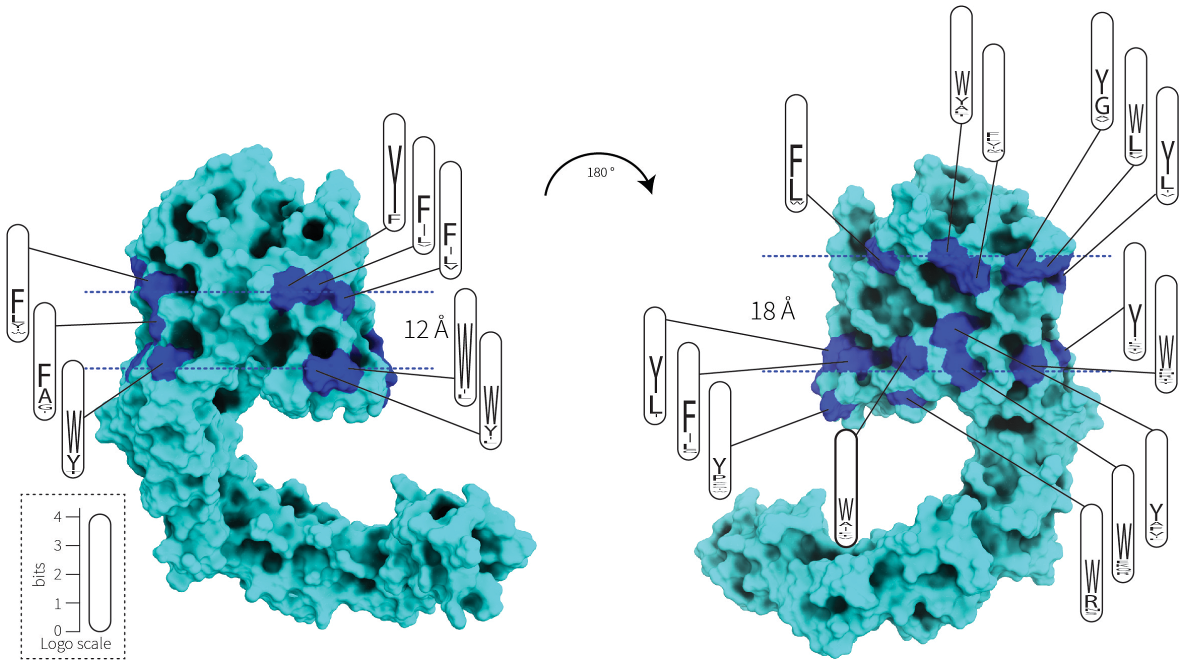

Supplementary Figure 1 | Placement and conservation of aromatic residues in TamA. Aromatic residues (dark blue) are more narrowly placed in this region of TamA than the 18-20 Å seen in other β-barrel proteins, as was reported for BamA[1]. The aromatic girdle of BamA is pinched across the face where the first and final β-strands of BamA meet, the last strand of BamA is only partly annealed with the first strand, and molecular dynamics simulations show movements of these β-strands that render the interior of the BamA barrel in the membrane plane more accessible to the lipid environment [1]. The aromatic girdle of TamA maps in a similar way to that found in BamA, pinched on one face to be less that the ~18-20 Å seen in other β-barrel proteins [1]. Blue-shading denotes the positions of the aromatic residues in TamA from *E. coli*, and each sequence logo denotes the relative abundance of residues found in those positions when considering the TamA sequences detailed in Supplementary Table S1. The relative height of the aromatic residue codes for tyrosine (Y), phenylalanine (F) or tryptophan (W) reflects the degree of conservation in each position. Aromatic residues form a girdle around β-barrel domains of other outer membrane proteins, indicative of the fixed positioning of lipid head groups in the bacterial outer membrane [2]. The Rate4Site analysis shows that the stringently conserved face of TamA is without a structurally restrictive aromatic girdle.

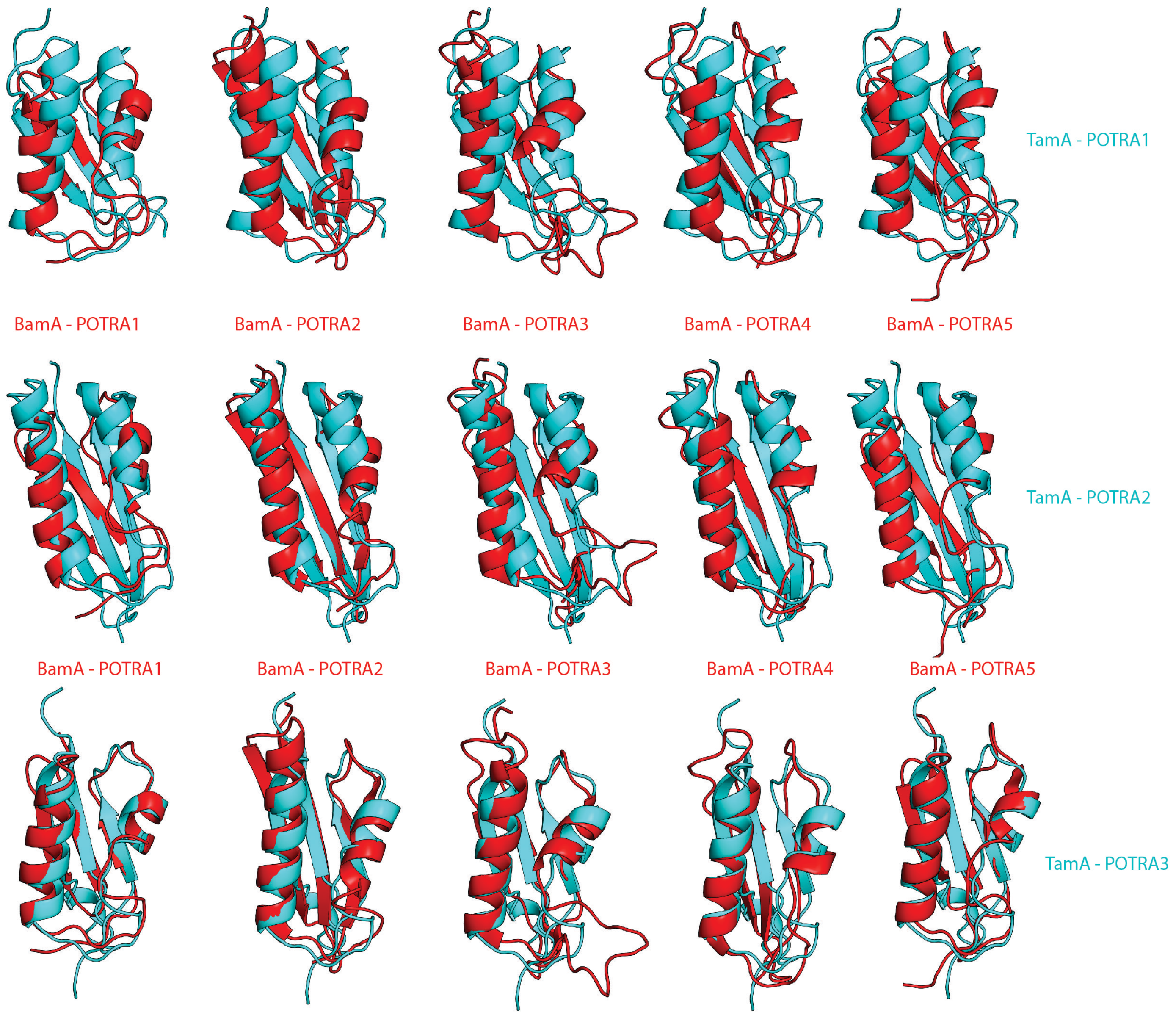

Supplementary Figure 2 | LSQ fits of TamA POTRA1, TamA POTRA2 and TamA POTRA3 with the BamA POTRA domains. Cartoon representation of the LSQ fits between TamA POTRA1 (cyan, top row), TamA POTRA2 (cyan, middle row) and TamA POTRA3 (cyan, bottom row) and the five BamA POTRA domains (shown in red) calculated using PyMOL (www.pymol.com/pymol). Neither TamA POTRA1 nor TamA POTRA2 have good structural agreement with the BamA POTRAs (see Supplementary Table S5) with no RMSD fit values lower than 3 Å, despite adopting the same general POTRA fold. The TamA POTRA3 domain is within the RMSD fit values previously calculated across all BamA POTRA domains (1.3-1.8 Å, see Supplementary Table S5). All the TamA POTRA domains also have a generally larger surface area than their BamA counter parts with an average surface area of 8500 Å compared to 7700 Å (Supplementary Table S5).

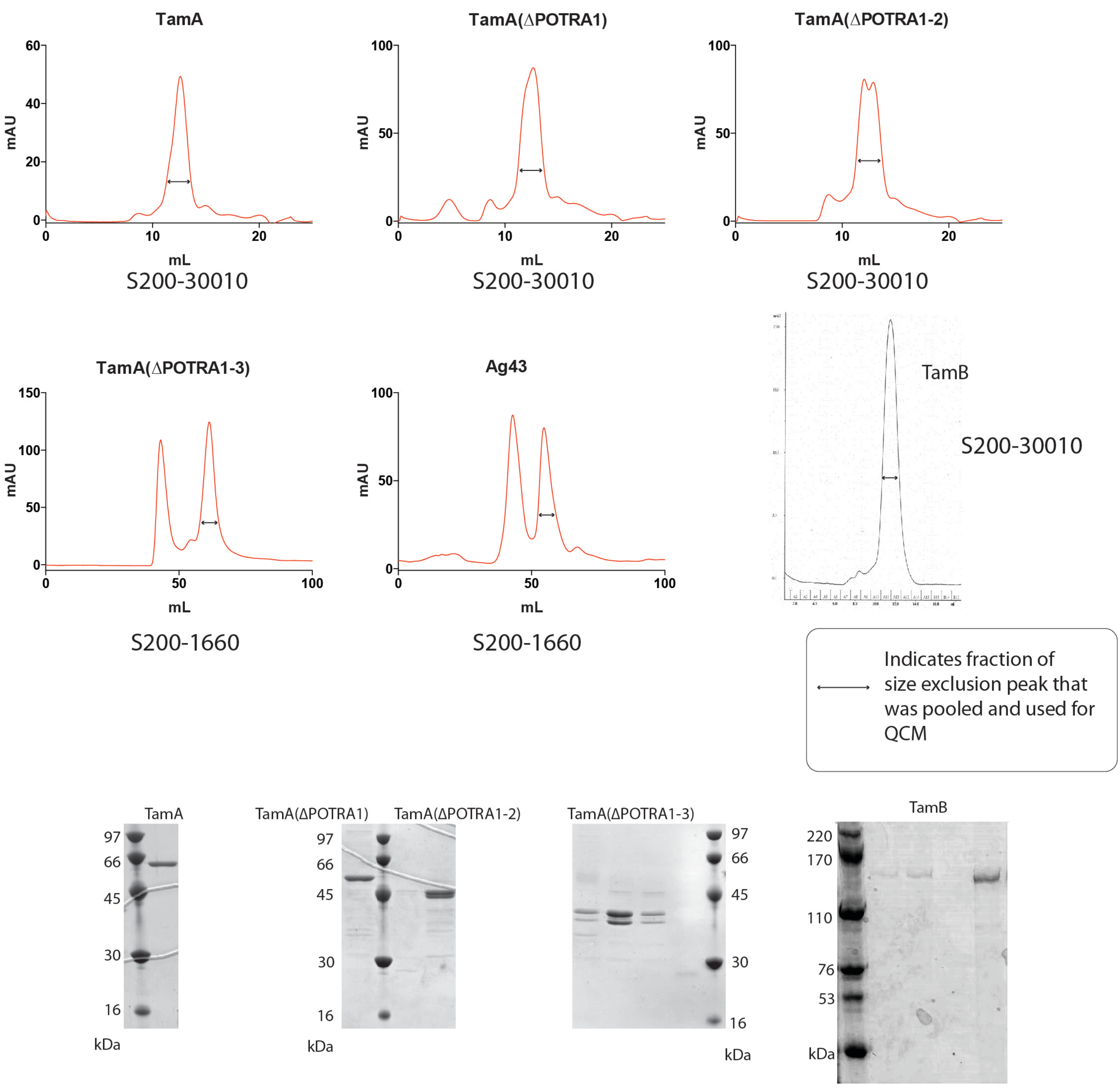

Supplementary Figure 3 | Protein purification for QCM-D. Size exclusion chromatography traces for the final stage of purification was undertaken with the indicated Sephadex columns (S200-30010 or S200-1660) as described in the Methods section. The chromatography profiles correspond to the elugent-solubilized membrane proteins TamA, TamA(ΔPOTRA1), TamA(ΔPOTRA1-2), TamA(ΔPOTRA1-3). Purification of the urea-denatured Ag43 was previously documented [3] and the size exclusion profile is shown here. In the size exclusion profiles routinely used to monitor protein quality, the fractions corresponding to the purified, monomeric protein are indicated with a double-headed arrow for the TamA constructs, TamB and Ag43. The purity of the fractions verified by SDS-PAGE, and Coomassie blue staining of gels are presented. The molecular weight standard sizes (kDa) are shown.

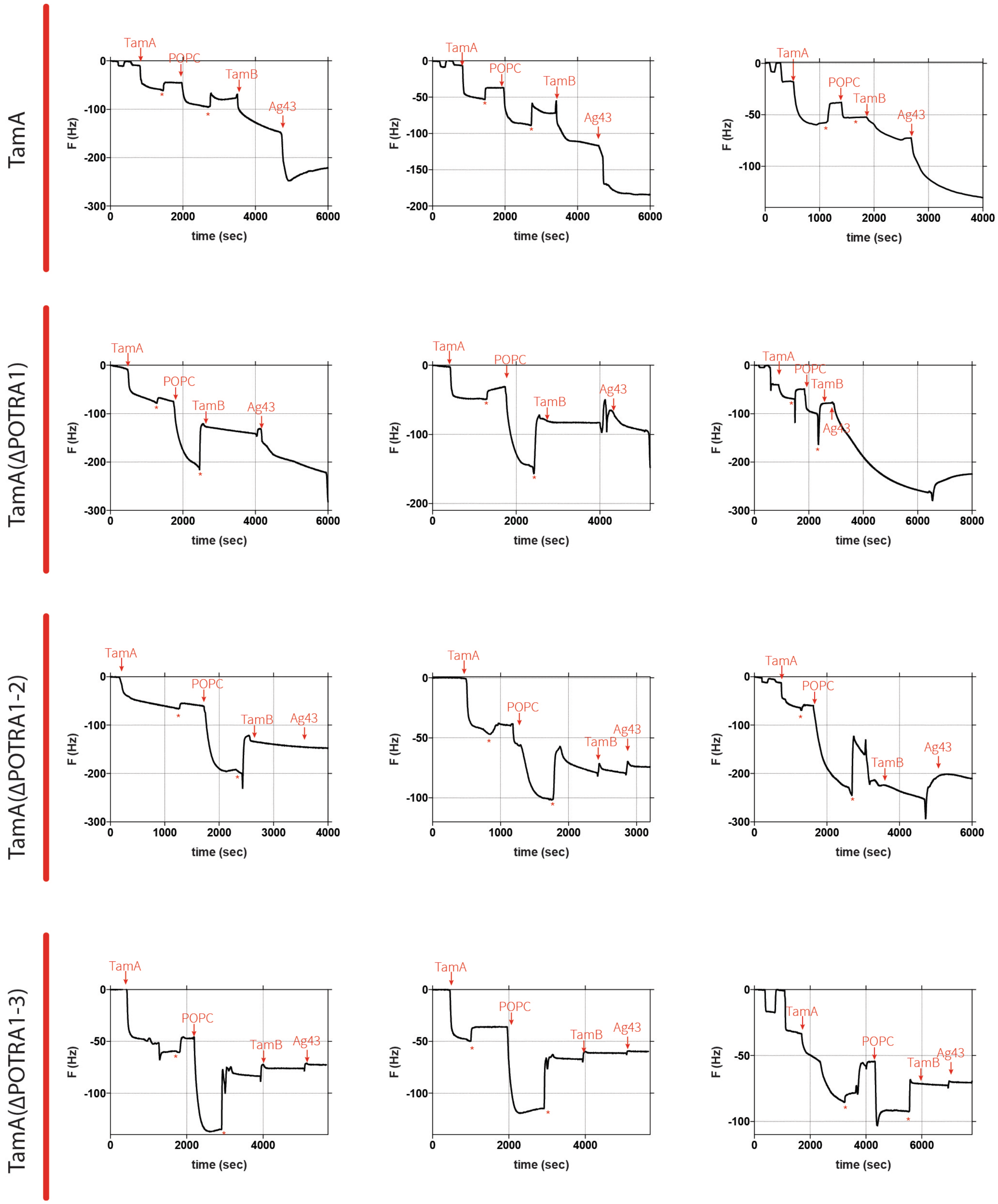

Supplementary Figure 4 | Analysis of TamA POTRA function by QCM-D. Replicate QCM-D measurements for the data shown in Figure 4. Shown is the frequency response, indicative of mass changes, to (1) the attachment of TamA (or TamA POTRA deletion, as indicated by row) to the gold-surface, (2) reconstitution of the membrane layer with phospholipids, (3) the addition of purified TamB revealing a frequency response of ~40Hz, and (4) addition of urea-denatured Ag43 revealing a frequency response of ~40Hz. Asterisks (*) – wash step (with 20mM TRIS and 150mM NaCl).

Supplementary Table 1 | Sequence accessions for TamA conservation analysis.

**Species**	**Family**	**TamA**
Escherichia coli str. K-12 substr. MG1655	Enterobacteriaceae	NP_418641.1
Shigella flexneri 2a str. 301	Enterobacteriaceae	NP_709978.1
Salmonella enterica subsp. enterica serovar Paratyphi A str. ATCC 9150	Enterobacteriaceae	YP_153277.1
Citrobacter rodentium ICC168	Enterobacteriaceae	YP_003366763.1
Citrobacter koseri ATCC BAA-895	Enterobacteriaceae	YP_001455125.1
Enterobacter cloacae subsp. cloacae ENHKU01	Enterobacteriaceae	YP_006576980.1
Klebsiella pneumoniae subsp. pneumoniae MGH 78578	Enterobacteriaceae	YP_001338234.1
Haemophilus haemolyticus M21639	Pasteurellaceae	EGT82363.1
Aggregatibacter actinomycetemcomitans ANH9381	Pasteurellaceae	YP_004948756.1
Pasteurella multocida 36950	Pasteurellaceae	YP_005177374.1
Mannheimia succiniciproducens MBEL55E	Pasteurellaceae	YP_087477.1
Coxiella burnetii RSA 493	Coxiellaceae	NP_819244.1
Vibrio fischeri MJ11	Vibrionaceae	YP_002155090.1
Photobacterium leiognathi subsp. mandapamensis svers.1.1.	Vibrionaceae	ZP_08312472.1
Grimontia hollisae CIP 101886	Vibrionaceae	ZP_06052894.1

Supplementary Table 2 | Sequences used in CLANS analysis.

**Species**	**TamA**	**BamA**
Shigella flexneri 2a str. 301	NP_709978.1	NP_706122.1
Salmonella enterica subsp. enterica serovar Paratyphi A str. ATCC 9150	YP_153277.1	YP_149572.1
Citrobacter rodentium ICC168	YP_003366763.1	YP_003363826.1
Coxiella burnetii RSA 493	NP_819244.1	NP_819641.1
Escherichia coli str. K-12 substr. MG1655	NP_418641.1	NP_414719.1
Haemophilus haemolyticus M21639	EGT82363.1	EGT83048.1
Pelobacter carbinolicus DSM 2380	YP_006715840.1	YP_006716969.1
Desulfobulbus propionicus DSM 2032	YP_004196024.1	YP_004194043.1
Geobacter sp. M18	YP_004199890.1	YP_004197490.1
Geobacter metallireducens GS-15	YP_006721763.1	YP_006721318.1
Desulfotalea psychrophila LSv54	YP_065180.1	YP_064017.1
Vibrio fischeri MJ11	YP_002155090.1	YP_002156782.1
Klebsiella pneumoniae subsp. pneumoniae MGH 78578	YP_001338234.1	YP_001333880.1
Enterobacter cloacae subsp. cloacae ENHKU01	YP_006576980.1	YP_006577378.1
Escherichia coli ETEC H10407	YP_006118110.1	YP_006113765.1
Citrobacter koseri ATCC BAA-895	YP_001455125.1	YP_001454710.1
Aggregatibacter actinomycetemcomitans ANH9381	YP_004948756.1	YP_004948333.1

Supplementary Table 3 | Parameters calculated from SAXS data.

	SAXS Data	X-ray Model (4C00)
$I(0)$ (cm^{-1})	$0.897\times10^{-2} \pm 0.554\times10^{-4}$	
R_{max} (Å)	91	90.4
R_g (Guinier) (Å)	27.7 ± 2.1	26.5
R_g ($P(r)$) (Å)	28.1 ± 0.2	
N.S.D.	0.66	

Supplementary Table 4| NMR structure statistics for the POTRA P1 domain.

Pairwise RMSD displacement (residues A22-V102)	
Backbone atoms (Å)	0.65 ± 0.14
Heavy atoms (Å)	1.46 ± 0.10
Non-redundant NOE distance restraints	
Total	1140 (14 per residue)
Intra (i = j)	344
Sequential (|i - j| = 1)	292
Short (|i - j| ≤ 5)	247
Long (|i – j| > 5)	257
Dihedral angle restraints	143
Hydrogen bond restraints	64 (2 per bond)
Deviations from experimental data	
NOEs (Å)	0.019 ± 0.001
Dihedrals (°)	0.242 ± 0.021
Deviations from ideal geometry	
Bonds (Å)	0.0021 ± 0.0001
Angles (°)	0.396 ± 0.008
Improper angles (°)	0.243 ± 0.014
Ramachandran statistics (residues A22-V102)	
Most favoured (%)	76.6
Additionally allowed (%)	21.6
Generously allowed (%)	1.8
Disallowed (%)	0.0

Supplementary Table 5 | LSQ structural alignments and area calculations of the TamA POTRA domain with BamA POTRA domains. Alignment type: Atom only = Super, Sequence alignment bias = CEAlign, as calculated by the PyMOL software package.(www.pymol.com/pymol). Models used for analysis; TamA-P1 (NMR Structure, this study), TamA-P2/TamA-P3 (Crystal Structure: 4C00), BamA-POTRAs (Crystal Structure: 4K3B).

Protein	Domain	Area (Å^2)
**TamA**	*P1*	8270
**TamA**	*P2*	8760
**TamA**	*P3*	8400
**BamA**	*P1*	6680
**BamA**	*P2*	7870
**BamA**	*P3*	9463
**BamA**	*P4*	6870
**BamA**	*P5*	7600

**Super – Pymol**					
RMS (Å)	**BamA-P1**	**BamA-P2**	**BamA-P3**	**BamA-P4**	**BamA-P5**
**TamA-P1**	3.035	2.661	3.445	2.774	3.382
**TamA-P2**	3.018	3.557	2.302	2.895	3.831
**TamA-P3**	1.802	3.884	3.038	2.092	1.520
	**BamA-P1**	**BamA-P2**	**BamA-P3**	**BamA-P4**	**BamA-P5**
**BamA-P1**		1.834	3.381	3.140	1.332
**BamA-P2**			1.676	1.779	3.683
**BamA-P3**				2.641	1.657
**BamA-P4**					3.179
**BamA-P5**					

**CEAlign – Pymol**					
RMSD (Å)	**BamA-P1**	**BamA-P2**	**BamA-P3**	**BamA-P4**	**BamA-P5**
**TamA-P1**	3.384	3.630	5.238	3.961	4.203
**TamA-P2**	3.525	2.552	4.001	3.372	3.694
**TamA-P3**	1.422	2.459	3.066	3.168	1.747
	**BamA-P1**	**BamA-P2**	**BamA-P3**	**BamA-P4**	**BamA-P5**
**BamA-P1**		2.270	3.080	2.953	1.288
**BamA-P2**			3.509	2.277	2.264
**BamA-P3**				3.631	3.026
**BamA-P4**					2.617
**BamA-P5**					

Supplementary Table 6 | Bacterial strains used in this study.

Strain	Description	Selectable markers	Source
MS1001	*Escherichia coli* "wild type" MG1655		
MS3944	*Escherichia coli* MG1655 with *tamAB* replaced by a kanamycin resistance (Kan^R) cassette	Kan^R	[4]
MS3994	MS3988 transformed with p*tamAB*	Kan^R, Chl^R, Amp^R	[4]
*tamA*Δ1 POTRA	MS3988 transformed with p*tamAB* -Δ1 POTRA (expression of protein TamA(ΔPOTRA1)	Kan^R, Chl^R, Amp^R	This study
*tamA*Δ2 POTRA	MS3988 transformed with p*tamAB* -Δ2 POTRA (expression of protein TamA(ΔPOTRA1-2)	Kan^R, Chl^R, Amp^R	This study
*tamA*Δ3 POTRA	MS3988 transformed with p*tamAB* -Δ3 POTRA (expression of protein TamA(ΔPOTRA1-3)	Kan^R, Chl^R, Amp^R	This study
E.coli T7 Express	NEB *E. coli* cell line containing λ DE3 prophage for expression of proteins from a T7 promoter		NewEngland Biolabs
E. coli BL21-DE3*	*Escherichia coli* cell line containing λ DE3 prophage for expression of proteins from a T7 promoter		Invitrogen
E. coli C41 Overexpress™	*Escherichia coli* cell line containing λ DE3 prophage for expression of proteins from a T7 promoter		Lucigen

Supplementary Table 7 | Plasmids used in this study

Plasmid	Description	Selectable markers	Source
pCM6 ($TamA_{POTRA}$)	pET-21d expressing *E. coli* MG1655 TamA POTRA domains (residues 22-293) under the control of a T7 promoter.	Amp^R	[4]
pMB1	pET-21d expressing *E. coli* MG1655 TamA N-terminal POTRA domain domain P1($TamA_{23\text{-}106}$) under the control of a T7 promoter.	Amp^R	This study
pMB16	pPROEx-HTb expressing *E. coli* MG1655 TamA N-terminal POTRA domains P1-3 ($TamA_{23\text{-}261}$) with a TEV protease cleavable Histadine purification tag.	Amp^R	This study
pSU2718	pACYC184 derivative broad host range cloning vector , IPTG inducible.	Chl^R	[5]
p*tamAB*	*tamAB* from *E. coli* MG1655 cloned into the XbaI-SphI site of pSU2718 under the control of the *lac* promoter. IPTG inducible.	Chl^R	[4]
p*tamAB* -Δ1 POTRA	pSU2718-*tamAB* with residues 22–101 of TamA deleted (expression of protein TamA(ΔPOTRA1)	Chl^R	This study
p*tamAB* -Δ2 POTRA	pSU2718-*tamAB* with residues 22–170 of TamA deleted (expression of protein TamA(ΔPOTRA1-2)	Chl^R	This study
p*tamAB* -Δ3 POTRA	pSU2718-*tamAB* with residues 22–250 of TamA deleted (expression of protein TamA(ΔPOTRA1-3)	Chl^R	This study
pBADTamAB-C-term-6xHis	pBAD24 arabinose inducible vector coding for TamAB with a 6x histadine tag inserted into loop 8 in TamA	Amp^R	[4]
pBADTamAB-C-term-6xHis-Δ1 POTRA	pBAD24 arabinose inducible vector coding for TamAB with a 6x histadine tag inserted into loop 8 in TamA with residues 22–101 of TamA deleted (expression of protein TamA(ΔPOTRA1)	Amp^R	This study
pBADTamAB-C-term-6xHis-Δ2 POTRA	pBAD24 arabinose inducible vector coding for TamAB with a 6x histadine tag inserted into loop 8 in TamA with residues 22–170 of TamA deleted (expression of protein TamA(ΔPOTRA1-2)	Amp^R	This study
pBADTamAB-C-term-6xHis-Δ3 POTRA	pBAD24 arabinose inducible vector coding for TamAB with a 6x histadine tag inserted into loop 8 in TamA with residues 22–250 of TamA deleted (expression of protein TamA(ΔPOTRA1-3)	Amp^R	[3]

Supplementary Table 8 | Primers used in this study.

Primer name	Sequence
For TamA.minusPOTRA3 (N-terminal POTRA)(deletes residues 22-101) (i.e. N-terminal POTRA)	TAAGCGGATCTGCCGTCGCC GTGCCGGTGTTAATTGGC
Rev TamA.minusPOTRA3(N-terminal POTRA) (deletes residues 22-101) (i.e. N-terminal POTRA)	GCCAATTAACACCGGCACGGCGACGGCAGATCCGCTTA
For TamA.minusPOTRA3&2 (N-terminal POTRA's) (Deletes from residues 22-170) (i.e. first 2 N-terminal POTRA)	TAAGCGGATCTGCCGTCGCC GCGCTCGGCCTGCATAAA
Rev TamA.minusPOTRA3&2 (N-terminal POTRAs) (Deletes from residues 22-170) (i.e. first 2 N-terminal POTRA)	TTTATGCAGGCCGAGCGCGGCGACGGCAGATCCGCTTA
ForTamA.minusPOTRA1-3. (i.e. all three POTRAs)	TAAGCGGATCTGCCGTCGCCACGAAAGTATTACCATTG
RevTamA.minusPOTRA1-3. (i.e. all three POTRAs)	CAATGGTAATACTTTCGTGGCGACGGCAGATCCGCTTA
2726	CGCCGCGAACGTCCGTCTACAGGTCGAGGGGTTATCGGGAC AGCTGGAAACACGTCTTGAGCGATTGTGTAGG
2727	TATAGCTTAGGCATCAGGCGATAACGTAACGTGAGTGTTGCT ATAGAGTCGACATGGGAATTAGCCATGGTCC
2729	TACATCCATGCACTCCCGTA
2730	TCCGTCAGGTACATTGGTCA
2732	ATATCTAGAAGAAAATGTGCGCTATATCC
2733	TTCGCATGCCTAAAACTCGAACTGATAGA
0214 – TamA(POTRA123) Fwd - NcoI	GCCGCCATGGCGAAGCTCCGTCTACAGG
MB114 – TamA(POTRA123) Reverse - XhoI	CCGCCTCGAGTCACGAAACCACGCCCGTC
0233a – TamA(P1) Fwd - NcoI	TTTGACGTCCATGGCGAACGTCCGTCTACAGGTCG
0233b – TamA(P1) Rev - XhoI	GTTGACGTCTCGAGCACGCCTGGCGTGACTTTG

SUPPLEMENTARY REFERENCES

1. Noinaj N, Kuszak AJ, Gumbart JC, Lukacik P, Chang H, Easley NC, Lithgow T, Buchanan SK (2013) Structural insight into the biogenesis of beta-barrel membrane proteins. *Nature* **501:** 385-390

2. Cowan SW, Schirmer T, Rummel G, Steiert M, Ghosh R, Pauptit RA, Jansonius JN, Rosenbusch JP (1992) Crystal structures explain functional properties of two *E. coli* porins. *Nature* **358:** 727-733

3. Shen HH *et al* (2014) Reconstitution of a nanomachine driving the assembly of proteins into bacterial outer membranes. *Nat Commun* **5:** 5078

4. Selkrig J *et al* (2012) Discovery of an archetypal protein transport system in bacterial outer membranes. *Nat Struct Mol Biol* **19:** 506-510, S501

5. Martinez E, Bartolome B, de la Cruz F (1988) pACYC184-derived cloning vectors containing the multiple cloning site and lacZ alpha reporter gene of pUC8/9 and pUC18/19 plasmids. *Gene* **68:** 159-162
